# Diagnosis of Hodgkin's Lymphoma Using Endobronchial Ultrasound-Guided Transbronchial Needle

**DOI:** 10.1155/2021/8910843

**Published:** 2021-02-27

**Authors:** Abdullah Fahad Almsareer, Ahmed Zaid Alkhathlan, Doaa Ali AlGhamdi, Khalid Alokla

**Affiliations:** ^1^Department of Medical Specialties, Pulmonary Division, King Fahad Medical City, Riyadh, Saudi Arabia; ^2^Department of Laboratory Medicine, Anatomical Pathology Division, King Fahad Medical City, Riyadh, Saudi Arabia

## Abstract

Endobronchial ultrasound-guided transbronchial biopsy has emerged as an excellent tool in diagnosing lung cancer. However, its use to diagnose lymphoma has been questioned, since the gold standard for diagnosing lymphomas is an excisional biopsy of involved lymph nodes. However, the procedure is sometimes risky or difficult. Recent studies have been showing great results using endobronchial ultrasound-guided transbronchial needle aspiration when accompanied by immunohistochemistry and cytology. Here, we present a case of Hodgkin's lymphoma patient that was accurately diagnosed using endobronchial ultrasound-guided transbronchial needle aspiration.

## 1. Introduction

Some of the most crucial features of a diagnostic procedure are high sensitivity, specificity, and overall diagnostic yield. Numerous studies have evaluated the effectiveness of endobronchial ultrasound-guided transbronchial needle aspiration (EBUS-TBNA) and have yielded different results concerning different aspects. A study carried by Tyan et al. yielded that EBUS-TBNA has nearly 89% diagnostic sensitivity [1]. In reference to this study, Nason and colleagues attribute to the real-time ultrasound within its set [2]. This has also facilitated sampling of smaller lymph nodes as well as a broader range of lymph node stations. Various studies have evaluated the effectiveness of EBUS-TBNA in the treatment of lymphomas, and in most cases, the result has been positive. Grosu et al. showed that EBUS-TBNA is useful in diagnosing and staging cancer and often included biomarkers analysis [3]. It has also shown great results regarding the diagnosis of lymphoma such as Hodgkin's lymphoma, and its more extensive use is attributed to the need for a less invasive endoscopic procedure while ensuring lower complication rates. We present a case of Hodgkin's lymphoma that was accurately diagnosed with the help of EBUS-TBNA.

## 2. Case Presentation

The patient is a forty-two-year-old man. He is a nonsmoker and with no known underlying medical conditions and had been referred from another healthcare facility to the pulmonary clinic having a history of chronic cough with sudden onset about a month ago. The cough was initially dry but later became productive of a mild-moderate amount of whitish sputum. The patient had about four to five episodes of hemoptysis in small amounts. He has no history of chest pain, wheezing, fever, or night sweating, but reports that he has lost about ten kilograms in four months and suffered a decrease in appetite.

**Figure 1:**
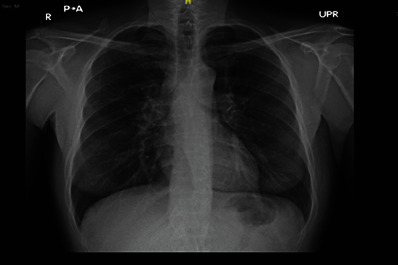
Chest X-ray showing bilateral hilar lymphadenopathy.

The patient's vitals were stable; however, the patient seemed underweight. There was no lymphadenopathy, splenomegaly, hepatomegaly, or skin rash. Other examinations were unremarkable. Laboratory tests are shown in Table 1. A chest X-ray was done, which showed bilateral hilar adenopathy (Figure 1).

**Figure 2:**
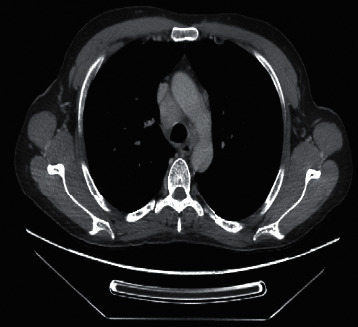
A CT scan of the chest (mediastinal window) showing enlarged right paratracheal lymph node measuring 18 mm in short axis.

A computed tomography (CT) scan was done at the other hospital, which revealed mediastinal and right hilar lymphadenopathy suspicious for malignancy (Figure 2). Focal areas of septal thickening in the right upper and middle lobe with no definite suspicious lung masses were noted. Tuberculosis work-up (acid-fast bacilli staining, culture, and nucleic acid amplification testing) was negative. Sputum for culture and sensitivity showed normal respiratory flora. A CT chest after IV contrast (compared with the CT chest from the other hospital) showed interval stability of the mediastinal and hilar lymphadenopathy. There was interval improvement in previously seen septal thickening in the right upper lobe and right middle lobes. Moreover, a CT of the abdomen and pelvis after IV contrast was done and showed subcentimetric lymph nodes in the gastrohepatic ligament.

**Figure 3:**
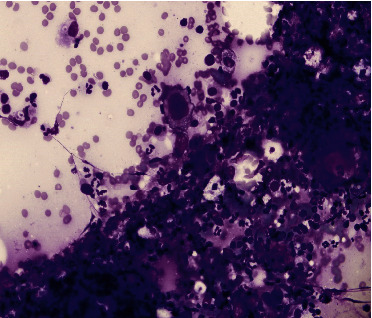
Right paratracheal lymph node biopsy via EBUS-TBNA showing Reed–Sternberg cells (Diff-Quick stain).

**Figure 4:**
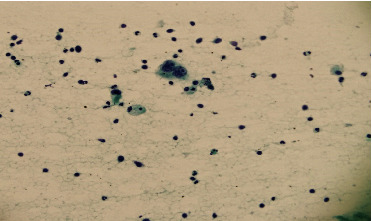
Right paratracheal lymph node biopsy via EBUS-TBNA with Papanicolaou stain showing Reed–Sternberg cells (×400).

**Figure 5:**
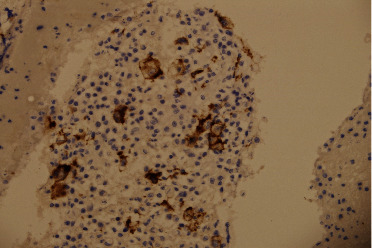
Right paratracheal lymph node EBUS-TBNA biopsy stained positive for CD30 immunochemistry (×40).

An EBUS-TBNA was done, and the right lower paratracheal lymph node (Station 4R) was sampled. The pathology revealed “scattered binucleated/multinuclear single large lymphocytes with prominent large macronuclei.” Moreover, a background of polymorphic lymphocytes and occasional bronchial cells were seen (Figure 3). The cytology testing showed findings that are highly suggestive of Hodgkin's lymphoma (Figure 4). Furthermore, the cell block revealed large atypical binucleated cells with positive CD30, CD15, and PAX5 (Figure 5). The cells are negative for CD20 and CD3. Standard PET of the whole body was performed and revealed diffuse mediastinal lymphadenopathy with large FDG uptake, consistent with lymphoma diagnosis. Spleen focal uptake is noted, which may indicate splenic involvement. Bone marrow involvement is highly suspected as well. The patient had a core biopsy of the mediastinal lymph node (mediastinoscopy), which revealed the same diagnosis. Therefore, the patient was diagnosed with Hodgkin's lymphoma stage IV SB, with a plan to start ABVD cycle chemotherapy and follow-up.

## 3. Discussion

Hodgkin's lymphoma is a curable malignancy if diagnosed early, with early intervention. Some of the options that are considered when choosing a diagnostic test include sensitivity, specificity, rate of complications, and overall diagnostic yield. Various studies have confirmed the effectiveness of EBUS-TBNA in diagnosing not only lymphoma but also lung cancer and other types of cancer, giving accurate results.

Some clinicians still argue that there is insufficient information to validate the speculated accuracy of EBUS-TBNA. They will opt for surgical excision and core biopsy as a sampling technique as the disease management is significantly based on the pathologic subtype and grade. Therefore, a few samples obtained through EBUS-TBNA may not be adequate. However, there are studies such as that conducted by Nason and colleagues, which shows that the real-time ultrasound incorporated in EBUS-TBNA has facilitated the sampling technique of this diagnostic method, thus rendering this claim unconvincing [2].

Similarly, developing data shows that EBUS-TBNA expertly diagnoses lymphoma when it is coupled with immunohistochemical, flowcytometric, and molecular studies and cytogenic studies. Grosu et al. showed that when EBUS-TBNA is used with immunohistochemistry and cytometry, it can give an overall of about 77% sensitivity and 100% specificity with a higher negative predictive value of 86% [3]. The study recommends an on-site assessment to improve the overall diagnostic yield of each case. The incorporation of triaging material during the EBUS-TBNA procedure to diagnose lymphoma warrants that there are sufficient materials for cytologic as well as ancillary studies, which were all needed in our patient. This is especially true when carrying out immunophenotyping on the biopsy. The latter can often strengthen the diagnostic yield of EBUS-TBNA and make its use appealing [1]. Triaging enables clinicians to make vital and accurate procedural decisions, for example, to increase passes during the procedure to maximize overall diagnostic yield. Furthermore, a systematic review and meta-analysis of 13 studies done by Labarca et al. showed the overall sensitivity and specificity of EBUS-TBNA were 66.2% and 99.3%, respectively. It also showed that ability of EBUS-TBNA to procure sufficient sample for subtyping lymphoma was 63% of positive samples. When this is augmented with immunophenotyping and flowcytometry, the sensitivity of EBUS-TBNA for diagnosing de novo and recurrent lymphoma improved to 72.4% and 80.5%, respectively [4]. The patient in this case study has mediastinal lymphadenopathy, which would necessitate work-up for lymphoproliferative conditions; therefore, it requires the use of immediate on-site assessment of such tools that will be employed here. Nason et al. supported this finding, confirming that rapid on-site evaluation, even though nondiagnostic, is appropriate during the procedure to permit better procedural decision making, and concluded that EBUS-TBNA has substituted most invasive procedures, such as mediastinoscopy, and still give definitive diagnosis within the setting [2]. Its high sensitivity and a lower rate of false-negative diagnosis for lymphoproliferative conditions, mainly when the sample is adequate, have provided an alternative that limits the use of mediastinoscopy.

Bandyopadhyay and colleagues mentioned that the main disadvantages of EBUS-TBNA included lesser sampling and inferior negative predictivity; however, there is more and more evidence supporting its use [5]. In one study, EBUS-TBNA was able to subclassify lymphoma in 87% of the cases [6]. There have been numerous advancements in technology that have overcome most of these, as shown by Grosu et al. [3]. EBUS-TBNA still diagnosed marginal zone and follicular lymphoma to reduce the increased level of discordance between a cytologic and histologic sample, which has been a concern in the past. The patient, in this case, had both histopathologic and cytologic samples.

## 4. Conclusion

The current advances in the minimally invasive diagnostic approach, particularly the EBUS-TBNA, including the on-site assessment, triaging samples, and flowcytometry, among others, have overcome the foregoing limitations that were holding EBUS-TBNA back. Currently, it is an effective tool for diagnosing lymphoma and can be used as the first line in lymphoma suspected cases with isolated mediastinal lymphadenopathy.

## Figures and Tables

**Table 1:** Laboratory results.

Lab test	Result	Normal value
WBC^a^	15 (×10^9^/L)	3.9–11 (×10^9^/L)
Neut%	84 (%)	30–70 (%)
Eos%	0.1 (%)	1–12 (%)
Lymph%	9.4 (%)	23–60 (%)
Hb^b^	14 (g/dL)	13.5–18 (g/dL)
Platelets	435 (×10^9^/L)	155–435 (×10^9^/L)
PT^c^	14 s	9.7–12.6 s
PTT^d^	37 s	25.3–38.3 s
INR^e^	1.1	0.81–1.23
Creatinine	50 (*µ*mol/L)	64–104 (*µ*mol/L)
Urea	3 (mmol/L)	3.2–7.4 (mmol/L)
Calcium	2.69 (mmol/L)	2.1–2.55 (mmol/L)
ESR	82 (mm/h)	0–20 (mm/h)
Albumin	42 (g/L)	35–52 (g/L)
Sodium	138 (mmol/L)	136–145 (mmol/L)
Potassium	3.73 (mmol/L)	3.5–4.5 (mmol/L)
Chloride	101 (mmol/L)	98–107 (mmol/L)
Bicarbonate	23 (mmol/L)	22–29 (mmol/L)

^a^White cell count. ^b^Hemoglobin. ^c^Prothrombin time. ^d^Partial thromboplastin time. ^e^International normalized ratio.

## Data Availability

Data used in this study are available on request by contacting the corresponding author, Abdullah Fahad Almsareer, via e-mail: aalmasareer@kfmc.med.sa.
